# Broadly neutralizing antibody responses in the longitudinal primary HIV-1 infection Short Pulse Anti-Retroviral Therapy at Seroconversion cohort

**DOI:** 10.1097/QAD.0000000000002988

**Published:** 2021-06-01

**Authors:** Luke A. Granger, Isabella Huettner, Franka Debeljak, Pontiano Kaleebu, Mauro Schechter, Giuseppe Tambussi, Jonathan Weber, Jose M. Miro, Rodney Phillips, Abdel Babiker, David A. Cooper, Martin Fisher, Gita Ramjee, Sarah Fidler, John Frater, Julie Fox, Katie J. Doores

**Affiliations:** a Department of Infectious Diseases, King's College London, Guy's Hospital, Great Maze Pond, London, UK; b Medical Research Council/Uganda Virus Research Institute, Entebbe, Uganda; c Projeto Praça Onze, Hospital Escola São Francisco de Assis, Universidade Federal do Rio de Janeiro, Rio de Janeiro, Brazil; d Department of Infectious Diseases, Ospedale San Raffaele, Milan, Italy; e Department of Medicine, Imperial College London, UK; f Infectious Diseases Service. Hospital Clinic–Institut d’investigacions Biomèdiques August Pi I Sunyer, University of Barcelona, Barcelona, Spain; g Peter Medawar Building for Pathogen Research, Nuffield Department of Medicine, University of Oxford, UK; h MRC Clinical Trials Unit at UCL, Institute of Clinical Trials & Methodology; i St Vincent's Centre for Applied Medical Research and The Kirby Institute, UNSW Australia, Sydney, NSW, Australia; j Brighton and Sussex University Hospitals, Brighton, UK; k HIV Prevention Research Unit, South African Medical Research Council, Durban, South Africa; l Department of Infectious Disease, Imperial College London; m NIHR Imperial Biomedical Research Centre, London; n Nuffield Department of Medicine, Oxford University; o Oxford NIHR Biomedical Research Centre, Oxford; p King's College NIHR Research Biomedical Research Centre, London, UK.

**Keywords:** broadly neutralizing antibody, HIV-1, longitudinal, vaccine

## Abstract

Development of immunogens that elicit an anti-HIV-1 broadly neutralizing antibody (bnAb) response will be a key step in the development of an effective HIV-1 vaccine. Although HIV-1 bnAb epitopes have been identified and mechanisms of action studied, current HIV-1 envelope-based immunogens do not elicit HIV-1 bnAbs in humans or animal models. A better understanding of how HIV-1 bnAbs arise during infection and the clinical factors associated with bnAb development may be critical for HIV-1 immunogen design efforts.

**Design and methods:**

Longitudinal plasma samples from the treatment-naive control arm of the Short Pulse Anti-Retroviral Therapy at Seroconversion (SPARTAC) primary HIV-1 infection cohort were used in an HIV-1 pseudotype neutralization assay to measure the neutralization breadth, potency and specificity of bnAb responses over time.

**Results:**

In the SPARTAC cohort, development of plasma neutralization breadth and potency correlates with duration of HIV infection and high viral loads, and typically takes 3–4 years to arise. bnAb activity was mostly directed to one or two bnAb epitopes per donor and more than 60% of donors with the highest plasma neutralization having bnAbs targeted towards glycan-dependent epitopes.

**Conclusion:**

This study highlights the SPARTAC cohort as an important resource for more in-depth analysis of bnAb developmental pathways.

## Introduction

A key goal in HIV-1 vaccine development is the elicitation of antibodies that potently neutralize a broad range of HIV-1 strains [1]. Such antibodies, termed broadly neutralizing antibodies (bnAbs), typically develop 2–3 years after infection in 10–30% of HIV-infected individuals [2–6], although in rare occasions can be detected within the first year of infection [7]. Although bnAbs are unable to control disease, when passively transferred to macaques, they can prevent infection in chimeric simian-HIV (SHIV) challenge models [8,9], highlighting the importance of eliciting a robust bnAb response in vaccine development. The virally encoded HIV-1 surface glycoprotein Env is the sole target for bnAbs and consists of a trimer of gp120-gp41 heterodimers. Several Env epitopes have been identified that are targeted by bnAbs isolated from chronically infected individuals. These include the CD4-binding site [10,11] (e.g. VRC01, N6), the membrane proximal external region (MPER) [12,13] (e.g. 4E10, 10E8), the fusion peptide [14,15] (e.g. ACS202, VRC34.01), and glycan-dependant epitopes centred around the N332-V3-region [16–18] (e.g. PGT121, PGT128, PCDN38A), the N160/V2-apex [16,19,20] (e.g. PG9, PGT145, CAP256-VRC26) and *N*-glycans at the gp120-gp41 interface [21,22] (e.g. PGT151, 35O22). HIV-1 bnAbs have unusual characteristics including high levels of somatic hypermutation, long CDRH3 regions, insertions and deletions and framework mutations [23]. bnAbs are thought to arise following multiple rounds of viral escape and antibody somatic hypermutation where the immune response is progressively targeted towards the most conserved regions of HIV-1 Env [24,25]. However, immunization with soluble recombinant state-of-the-art Env trimers has so far not generated antibodies capable of neutralizing a broad range of HIV-1 isolates [26–28]. Therefore, understanding how bnAbs arise during natural infection, and the clinical and virological correlates relating to their elicitation, may be critical for the development of immunogens capable of eliciting protective bnAbs through vaccination.

As clinical guidelines have moved towards early antiretroviral therapy (ART) following diagnosis [29,30], there is great value in historic longitudinal clinical cohorts for studying bnAb development. Therefore, we set out to phenotype bnAb development in individuals diagnosed with primary HIV-1 infection and randomized to the control arm of the Short Pulse Anti-Retroviral Therapy at Seroconversion (SPARTAC) trial [31]. The SPARTAC trial was initiated in 2003 to determine whether short-term ART during primary HIV-1 infection (either 12 or 48 weeks) could lengthen the time before long-term ART was required. Individuals were recruited to the trial within 6 months of HIV-1 infection. Within this trial, one-third of 376 study participants were randomly allocated to no immediate ART, which reflected standard clinical practice at the time. Longitudinal plasma and peripheral blood mononuclear cells (PBMCs) were collected sequentially until CD4 levels declined to 350 cells/μl and ART was initiated, or conclusion of the trial (which ever came first). Participants were recruited in the UK, Ireland, South Africa, Uganda, Italy, Spain, Brazil and Australia, and therefore included multiple HIV-1 clades. Using clinical samples from participants enrolled into the control arm, we set out to: identify donors that developed bnAbs, identify clinical factors that associate with bnAb induction, study kinetics of bnAb development and study specificity of bnAb responses.

Plasma taken either just prior to initiation of ART or just prior to termination of the trial from 8 of 50 donors (16%) in the control arm were found to neutralize at least five viruses from a cross-clade 6-virus indicator panel [4]. Development of neutralization breadth and potency was shown to strongly correlate with higher viral loads and longer infection times. In the donors with highest breadth and potency, heterologous neutralization appeared between 50 and 108 weeks post infection and neutralization breadth typically took 132–204 weeks to develop and arose in a stepwise manner. BnAb activity was mostly directed to one or two epitopes per donor with more than 60% of donors with neutralization scores more than 0.9 having bnAbs targeted towards glycan-dependent epitopes. This study highlights the SPARTAC cohort as an important resource for more in-depth analysis of bnAb developmental pathways that can inform HIV-1 immunogen design strategies.

## Methods

### Cohort design and ethics

The design of the SPARTAC trial is reported elsewhere [31]. SPARTAC was an international randomized control trial of early ART, comparing 12 or 48 weeks of ART followed by treatment interruption with no immediate treatment. Three hundred and sixty-six adults within an estimated 6 months from seroconversion were recruited. All participants gave written informed consent. Ethical approval for collection of these samples was originally approved by: Medicines and Healthcare products Regulatory Agency (UK), Ministry of Health (Brazil), Irish Medicines Board (Ireland), Medicines Control Council (South Africa) and The Uganda National Council for Science and Technology [31].

### Reagents

The following reagents were obtained through the NIH AIDS Reagent Program: HIV-1 RSC3 (Cat#12042) and HIV-1 RSC3 Δ371I/P363N (Cat#12362) recombinant proteins from Drs Yang, Kwong, Nabel [32,33], and HIV-1 Consensus B MPER Peptide (Cat#11938).

### Env-pseudovirus production and neutralization assays

HIV-1 pseudovirus was generated in HEK-293T cells as described [6,34]. HEK-293T cells were transfected with plasmids expressing the HIV-1 virus backbone PSG-3ΔEnv and full-length Env at a ratio of 2 : 1 using polyethylenimine (PEI) (1 mg/ml; 1 : 3 PEI/total DNA; Polysciences, Warrington, Pennsylvania, USA). Virus supernatants were harvested after 72 h.

Plasma neutralizing activity was assessed using TZM-bl target cells (expressing CD4, CXCR4 and CCR5 receptors) as described previously [6,34]. TZM-bl cells were seeded 24 h in advance in a 96-well plate (10 000 cells/well). Heat inactivated plasma (56 ^o^C, 1 h) was serially diluted and preincubated with virus for 1 h before addition to the cells. Luminescence was quantified after 72 h via lysis and addition of Bright-Glo luciferase substrate (Promega, Madison, Wisconsin, USA). ID_50_ was determined using nonlinear regression (GraphPad Prism).

### Calculation of neutralization score

The cross-clade indicator panel included JR-CSF (clade B), 94UG103 (clade A), 92RW020 (clade A), IAVI C22 (clade C), 92TH021 (clade CRF01_AE) and 92BR020 (clade B) [4]. VSV-G was used as a control for nonspecific neutralization. Neutralization breadth and potency of individual plasma samples were determined by a neutralization score defined as the weighted average of log-transformed ID_50_ values across the cross-clade virus panel: [score = average (log3 (dilution/100) + 1)] as previously described [24]. All titres below the limit of detection (1 : 50) were assigned a value of 33 for purposes of calculating a neutralization score. Log-transformed values with 0.0 represent a sample with undetectable neutralization.

### Serum mapping by neutralization

*N*-glycan sites were removed through an Asn to Ala or Asn to Lys mutation in the consensus sequence (NXT/S) as previously reported [35]. Variant viruses were selected based on plasma neutralization activity and included; JR-CSF (N332A, N160K, N611A + N637A), IAVI C22 (N160K, N332A, E647A), 92TH021 (N334A), 94UG103 (N160K, N332A), 92RW020 (N160K, N332A, N611A + N637A), 92BR020 (N160K, N332A).

### Membrane proximal external region peptide and RSC3 competition neutralization assay

Patient plasma was preincubated with either MPER peptide (50 μg/ml) or RSC3 glycoprotein (25 μg/ml) for 1 h before addition of HIV-1 pseudovirus. The plasma/MPER or plasma/RSC3 pseudovirus mixture was incubated for a further 1 h before addition of TZM-bl cells. Neutralization was determined as described above.

### RSC3 ELISA

Wells were coated with 50 ng of RSC3 or RSC3 mutant (RSC3Δ371I/P363N) [32,33] overnight (4 °C). Plates were washed five times with PBS-containing 0.05% Tween20 (PBS-T) and blocked with blocking buffer (5% nonfat milk in PBS-T) for 1 h (RT). Serial diluted plasma (starting at 1 : 50) was incubated for 2 h (RT). Plates were washed five times with PBS-T. Secondary Ab (goat-antihuman F(ab’)_2_-AP, (Invitrogen, Paisley, UK, Cat# 31312, 1 : 1000) was added for 1 h and plates washed. p-Nitrophenyl phosphate substrate (Sigma-Aldrich, Gillingham, Dorset, UK) was added and optical denisty measured at 405 nm.

### Statistics

Statistical analyses were performed using GraphPad Prism 8; GraphPad Software, San Diego, California, USA). Differences between groups were analysed with the Mann--Whitney *U* test or ANOVA. Spearman's rank correlation was used to examine the associations between the nonparametric factors studied. Principle component analysis was performed using R, R studio and the package FactoMineR. Univariate and multivariate linear regression analyses were performed using GraphPad Prism and R. For multivariate analysis, neutralization score was used as dependent factor and weeks post recruitment, start time of ART and logarithmic viral load at recruitment and neutralization score were used as potential predictors. The two parameters with the highest *P* value (start time of ART and logarithmic viral load at recruitment) were further eliminated and tested in multivariate analysis using neutralization score as dependent factor. *P* values less than 0.05 were considered significant.

## Results

### Broadly neutralizing antibody responses in the participants randomized to the Short Pulse Anti-Retroviral Therapy at Seroconversion cohort control arm

**Fig. 1:**
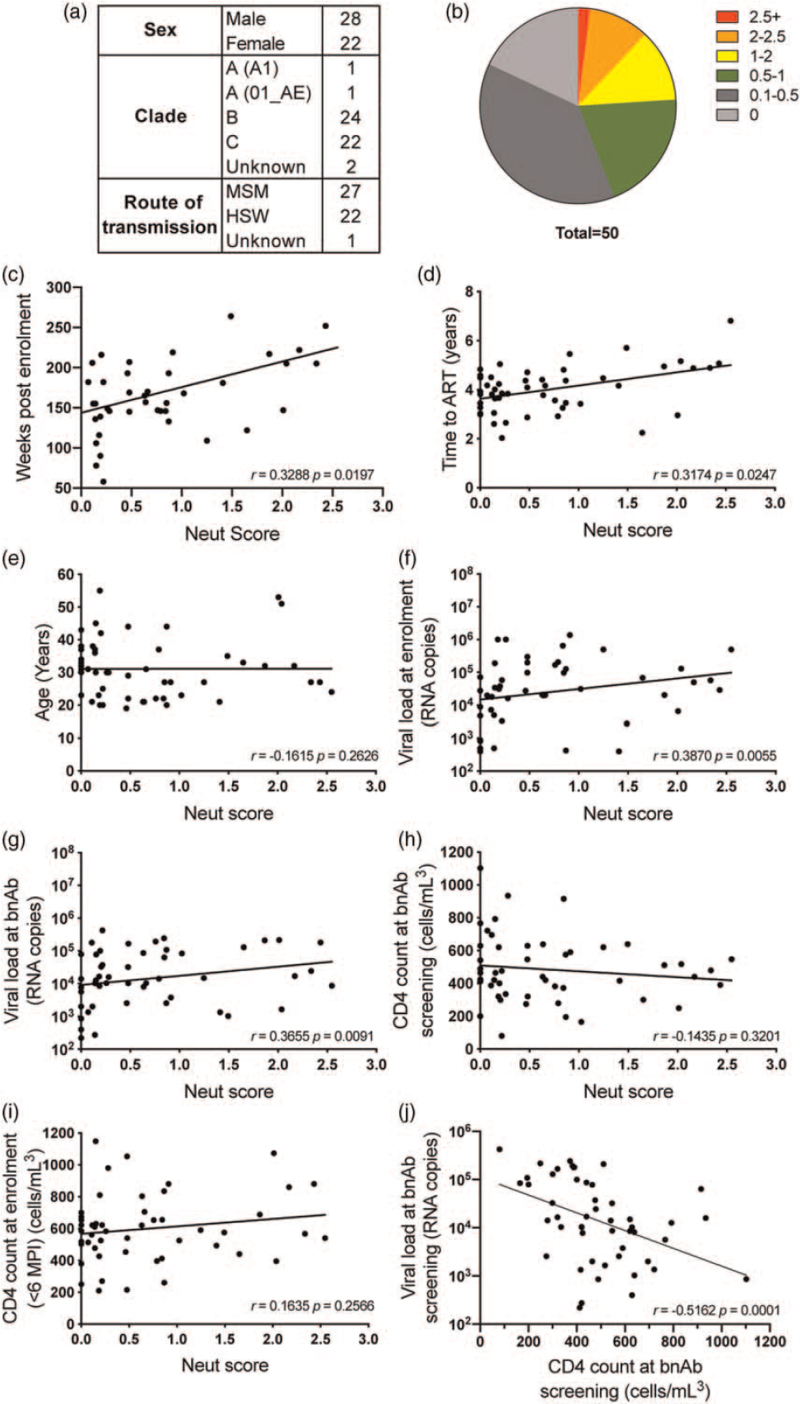
Neutralizing antibody responses in the SPARTAC cohort.

Fifty individuals from the SPARTAC trial control arm were selected based on availability of biobanked plasma samples (Fig. 1a). Individuals came from study sites in the UK (*n* = 18), South Africa (*n* = 21), Brazil (*n* = 2), Australia (*n* = 4) and Italy (*n* = 5) and 56% of this cohort were male. Neutralization breadth and potency of plasma at the time point prior to initiation of ART or termination of the trial (median 157.5 weeks post enrolment) were determined using a cross-clade 6-virus indicator panel (JR-CSF, 92BR020, 92RW020, 94UG103, IAVI-C22, 92TH021) previously shown to be predictive of breadth and potency on a much larger virus panel [4,18]. Neutralization of virions pseudotyped with vesicular stomatitis virus glycoprotein (VSV-G) was used to determine nonspecific neutralization. The plasma dilution required to reduce the level of infection below 50% (ID_50_) was calculated and used to assign each patient a neutralization score representing both breadth and potency against the indicator panel (Table S1) [36]. One donor (2%) had a neutralization score greater than 2.5 and five donors (10%) had a neutralization score greater than 2.0 (Fig. 1b). Nine donor plasma samples (18%) showed no neutralizing activity on the viruses tested. This frequency of bnAb activity is similar to that previously reported for other HIV-1 infection cohorts [3,24,36–39].

(a) SPARTAC cohort demographics. (b) Plasma from the control arm of the SPARTAC trial were collected and the neutralization breadth and potency measured on a cross-clade HIV-1 indicator panel. Neutralization scores were calculated as described in the methods section. Percentage of donors with neutralization scores at defined cut-offs are shown in the pie-graph. Correlation between clinical parameters and neutralization score for (c) weeks post trial enrolment, (d) years to initiation of ART, (e) age of participant at enrolment, (f) viral load at trial enrolment, (g) viral load at the time at which neutralization score was measured, (h) CD4^+^ T-cell count at the time at which neutralization score was measured, (i) CD4^+^ T-cell count at less than 6 months post infection. (j) Correlation between CD4 count at the time at which neutralization score was measured and viral load. Correlations were assessed by Spearman analyses using GraphPad Prism 8: *P* values and *r* values are indicated. Linear, or semi-Log regressions are shown as black lines. ART, antiretroviral therapy; SPARTAC, Short Pulse Anti-Retroviral Therapy at Seroconversion cohort.

### Clinical factors associated with development of broadly neutralizing antibodies in the Short Pulse Anti-Retroviral Therapy at Seroconversion cohort

Next, we conducted statistical analyses to identify potential associations between bnAb development and clinical factors recorded during the trial (Fig. 1c--h). Similar to previous observations [36,37], the duration of HIV-1 infection strongly correlated with neutralization score (Fig. 1c, Spearman *r* = 0.3325, *P* = 0.0183). There was also a positive correlation between time to initiation of ART (a reflection on duration of infection) and neutralization score (Fig. 1d, Spearman *r* = 0.3147, *P* = 0.0247). There was no correlation with the age (all of whom were >18 years) and neutralization score (Fig. 1e). Neutralization score correlated with higher viral loads at both trial enrolment (Fig. 1f, Spearman *r* = 0.3870, *P* = 0.0055) and time of bnAb measurement (Fig. 1g, Spearman *r* = 0.3655, *P* = 0.009). Unlike previous studies, which found lower CD4^+^ T-cell count were associated with bnAb development [3,36,40,41], there was no correlation between CD4 levels at either enrolment or at the timepoint at which neutralization was measured, and neutralization score within the SPARTAC cohort (Fig. 1h and i). High viral loads did correlate with low CD4 counts at the time of bnAb screening (Fig. 1j).

**Fig. 2:**
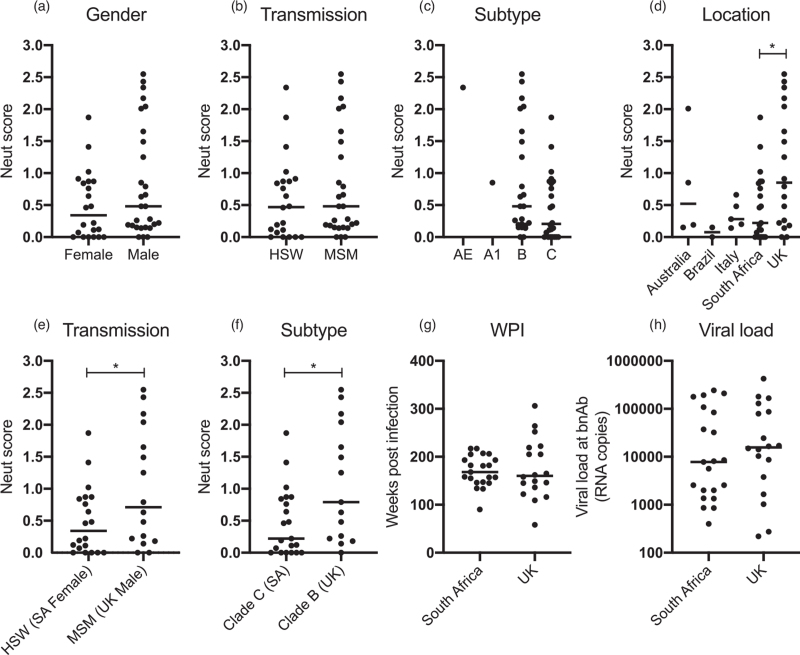
Factors relating to neutralization score.

Neutralization score was not associated with HIV-1 subtype, route of transmission or gender (Fig. 2a and b). There were higher neutralization scores in clade B compared with clade C infected donors but this did not reach statistical significance (Fig. 2c). However, there was a significantly higher neutralization score in UK donors compared with those from South Africa (Fig. 2d), with all South African donors being female individuals. The small group sizes for Italy, Brazil and Australia did not allow meaningful analysis. To understand factors that might contribute to the higher neutralization scores in UK donors, we carried out further analysis comparing donors from South Africa and the UK only (Fig. 2e--i). South African participants were mostly women infected with clade C viruses through heterosexual transmission (HSW) whilst UK participants were mostly MSM infected with clade B viruses. Statistically significant higher neutralization scores were observed in male UK MSM donors compared with female South African HSW donors (Fig. 2e) and in UK Clade B infection compared with South African Clade C infection (Fig. 2f). However, these differences were not because of duration of HIV infection (Fig. 2g) or increased viral load (Fig. 2h) in the UK donors. Whilst multiple factors were correlated with neutralization score whenever analysed individually, in a multivariate analysis of significant linear correlated predictors, only viral load at bnAb measurement and weeks post recruitment were shown to predict the neutralization score independently (Table S2). After the model was adjusted to contain only weeks post recruitment and viral load at neutralization score, both were still independent predictors.

Differences in neutralization score across the whole SPARTAC cohort for: (a) gender, (b) transmission route [MSM and men to women heterosexual transmission (HSW)], (c) subtype, and (d) country samples were collected. For (d) an ANOVA analysis showed no significant difference in neutralization score between the countries. However, a Mann--Whitney *U* test showed a significantly higher neutralization score in UK vs. South African participants (*P* = 0.0247). Difference in neutralization scores for UK and South African participants only for: (e) gender and transmission route (MSM from UK and HSW women from South Africa) (*P* = 0.0370), (f) subtype (*P* = 0.0266), (g) weeks post trial enrolment, and (h) viral load at bnAb measurement. Statistically significant differences between groups were determined using Mann--Whitney *U* test and the respective *P* values are shown. The horizontal bars represent the median values for each group. ANOVA, analysis of variance; SPARTAC, Short Pulse Anti-Retroviral Therapy at Seroconversion cohort.

### Specificity of broadly neutralizing antibody responses in the SPARTAC cohort

Next, we mapped the specificity of the bnAb responses in individuals with neutralization scores above 0.9 (Table 1 and Tables S3–S6). Firstly, N332/V3, V2-apex and interface specificity were determined using neutralization assays on pseudoviruses where either the N332, N160 and N611/N637 glycan sites, respectively, had been deleted using an asparagine to alanine or lysine mutation. Four donors (31%) showed a reduction in neutralization sensitivity across multiple viruses for the N332A mutation (3–300-fold), three donors (23%) showed reduction for the N160A/K mutations (8–81-fold), and three donors (23%) showed bnAb specificity against the quaternary epitope at the gp41/gp120 interface (5–400-fold) (Table S3). In some cases, an enhancement in neutralization potency was observed when a glycan site was removed, in particular, the N160 glycan (Table S3). As neutralizing activity was not decreased with glycan mutations for all the viruses tested, nAbs with narrower breadth may also be present that contribute to the plasma neutralization.

**Table 1:** Specificity of bnAbs present in SPARTAC donors with neutralization score greater than 0.9.

		CD4-binding site				MPER	N160	N332	Interface	
Donor ID	Neut Score	RSC3 ELISA (CD4-bs)	RSC3 comp (CD4-bs)	RSC3 ELISA (equal binding)	RSC3 comp (equal competition)	MPER comp	N160A/K	N332A	N611A/N637A	Predominant epitope
SUM036008	2.55	–	n.d.	+	n.d.	–	+++	–	–	N160
SUT036022	2.43	–	n.d.	++	n.d.	–	–	+	–	N332
SUV054003	2.34	–	n.d.	++	n.d.	–	–	–	–	Unknown
SUP033003	2.17	+	–	–	–	+	–	–	–	Mixed
SUV214008	2.04	–	n.d.	–	n.d.	–	–	+++	–	N332
SAR032004	2.01	++	–	–	n.d.	–	–	–	–	CD4 (dRSC3)
SJU027003	1.87	+/−	–	–	+	–	–	+++	–	Mixed
SUM036079	1.65	–	–	–	–	–	+	–	+	Mixed
SUF214003	1.49	–	n.d.	–	n.d.	–	+	–	–	N160
SJA023027	1.41	+/−	–	–	–	–	–	–	–	CD4 (dRSC3)
SUW036083	1.25	+/−	–	–	–	+	–	–	–	Mixed
SJE023008	1.02	–	n.d.	–	n.d.	+	–	+	+	Mixed
SUT033001	0.91	–	n.d.	–	n.d.	−	–	–	+	Interface

To determine the Env region targeted by the bnAbs present in patient plasma serum, mapping analysis was performed. The symbols represent the strength of the bnAb phenotype observed. RSC3 (CD4 bs): If the ratio between the area under the curve (AUC) for RSC3/RSC3Δ371I P363N is 2–3 (+/−), 3–8 (+) and greater than 8 (++). RSC3 competition (CD4 bs): + if the neutralization is decreased by at least three-fold for RSC3 but not RSC3Δ371I/P363N. Only those plasma with binding to RSC3 were tested in the RSC3 competition neutralization. RSC3 (equal binding): if the ratio between AUC of RSC3/RSC3Δ371I/P363N) is 1.5 or less and the binding level is as a percentage of the 2G12 endpoint titre as follows; ++ at least 50%, + is less than 50%, but at least 25%, +/− is 25% or less. RSC3 competition (equal competition): + if the neutralization is decreased by ≥3 fold for both RCS3 and RSC3Δ371I/P363N. MPER: + indicates a decrease in plasma neutralization potency of at least three-fold when competed with soluble MPER peptide. N332A, N160A/K and interface epitopes: + indicates neutralization of one virus decreased three-to-five fold, ++ indicates at least two viruses decreased three-to-five fold, +++ indicates at least five-fold decrease for at least two viruses with glycan site deletion. n.d. means not determined. SPARTAC, Short Pulse Anti-Retroviral Therapy at Seroconversion cohort. The data relating to this table can be found in Tables S3 and S6.

MPER specificity was determined using a competition for neutralization assay with a synthetic MPER peptide. Plasma was preincubated with MPER peptide before addition of virus. A decrease in neutralization compared with no MPER peptide was seen for three donors (23%) (Table S4). CD4 specificity was assessed using the resurfaced stabilized gp120 Core 3 (RSC3), a protein that is selective for VRC01-like CD4-binding site bnAbs, and a CD4-binding site knock out mutant (ΔRSC3, RSC3Δ371I P363N) that has decreased binding for VRC01-like bnAbs [32,33]. Of the 13 donors studied, 5 donors (38%) showed differential binding between the RSC3 wild-type and RSC3Δ371I P363N mutant to some extent (Table 1 and S5) (although 2 donors had very low endpoint titres). However, similar to previous studies [32,36], neutralization was not competed by soluble RSC3 (Table S6) suggesting that although RSC3 reactive Abs can be found in 40–60% of HIV-1-infected individuals, the RSC3 reactive Abs are not mediating the broad neutralization activity. Nevertheless, three of these donors showed neutralization directed against another bnAb epitope. Three donors (23%) showed equally strong reactivity to both RSC3 and ΔRSC3. As this probe was engineered to display only the CD4-binding-site epitope, and as PGT128 and 2G12 bound to both probes indiscriminately (data not shown) [32], we reasoned that this phenotype could either be representative of a V3-glycan epitope or 2G12-like or a CD4-binding site bnAb, such as N6, which shows no differential binding to RSC3 and ΔRSC3 [11].

Overall, seven donors (55%) had bnAb responses predominantly targeting a single neutralizing epitope, five donors (38%) had nAbs targeting multiple known bnAb epitopes and one donor (8%) had undefined specificity. bnAbs induced against the N332/V3 epitope were most prevalent in this cohort at the timepoints analysed.

### Kinetics of broadly neutralizing antibody development in the Short Pulse Anti-Retroviral Therapy at Seroconversion cohort

**Fig. 3:**
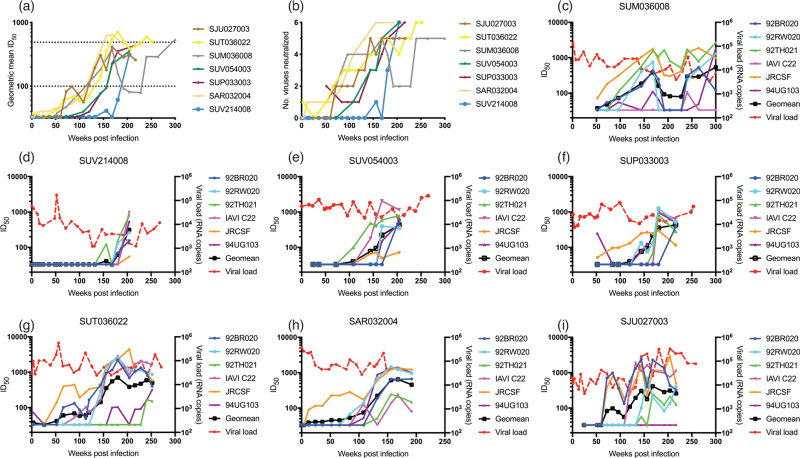
Longitudinal analysis of broadly neutralizing antibody development for SPARTAC donors with the highest neutralization scores.

Using longitudinal plasma samples, we measured the kinetics of bnAb development for the seven donors with the highest neutralization scores (Fig. 3). In all donors, there was a delay in development of heterologous neutralization following HIV-1 infection, and the pattern and timing of bnAb development differed between donors (Fig. 3a and b). For example, heterologous neutralization in donor SUV214008 arose late in infection, after 180 weeks but the bnAbs that arose neutralized all viruses in the panel to some extent (Fig. 3g). In contrast, heterologous neutralization activity started to appear after 50 weeks in donor SJU027003 but the neutralization breadth took time to develop, increasing incrementally to reach neutralization of five viruses after 160 weeks (Fig. 3i). The geometric mean titres peak in some donors (e.g. SJU027003) with little or no increase in titres thereafter. Development of bnAbs had no effect on viral load in any of the donors (Fig. 3c--i).

Neutralization by plasma over multiple time-points was measured for each donor on the cross-clade HIV-1 indicator panel. (a) The geometric mean across the six-virus panel was calculated at each time point for each donor as described in the methods. (b) Number of viruses neutralized with an ID_50_ greater than 50 at each time point is shown for each donor. ID_50_ values for neutralization of individual viruses at each time point (left axis) as well as geometric mean ID_50_ across the indicator panel (black line) and viral load (right-axis, red dotted line) for (c) SUM036008, (d) SUT036022, (e) SUV054003, (f) SUP033003, (g) SUV214008, (h) SAR032004 and (i) SJU027003.

**Fig. 4:**
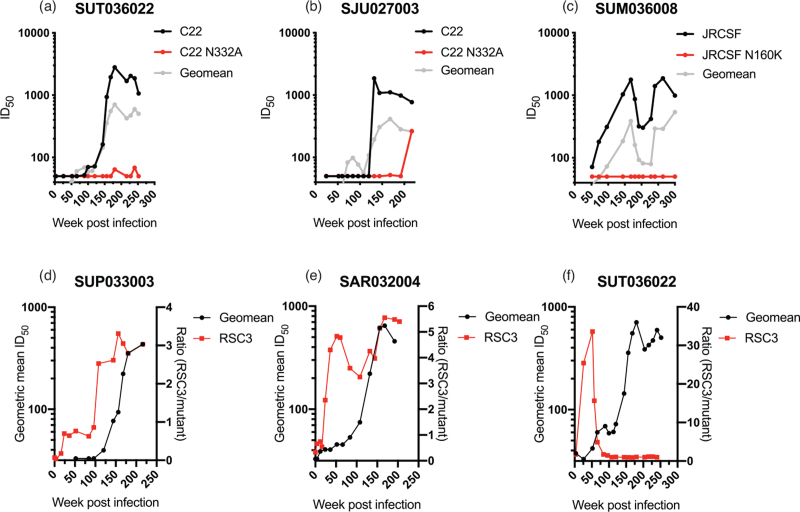
Longitudinal epitope mapping.

We further determined the bnAb specificity over time in some donors using longitudinal plasma samples (Fig. 4). For donors with an N332/V3 phenotype (SUT036022 and SJU027003) or N160 phenotype (SUM036008), nAbs sensitive to the N332A mutation or N160K mutation arose at the same timepoint as heterologous neutralization (Fig. 4a--c). However, donors with a CD4-binding site phenotype (SUP033003 and SAR032004) had CD4-binding site-specific Abs present before heterologous neutralization arose (Fig. 4d and e, respectively). This observation, combined with the lack of RSC3 competition for neutralization, further suggests this epitope is not responsible for the broadly neutralizing activity in plasma from these donors. Interestingly, donor SUT036022, which had strong binding to both the RSC3 wild-type and mutant glycoproteins at late infection timepoints, showed a CD4-binding-site phenotype preceding the development of heterologous neutralization with N332/V3 bnAb specificity, which disappeared later in infection (Fig. 4f).

(a and b) Neutralization of IAVI C22 wild-type (black line) and N332 glycan-site deleted variant virus (N332A) (red line) compared with the geometric mean ID_50_ titre (grey line) for SUT036022 and SJU027003, respectively. (c) Neutralization of JR-CSF wild-type (black line) and N160 glycan-site deleted viruses (N160K) (red line) compared geometric mean ID_50_ titre (grey line) for SUM036008. (d and f) Fold-change in area under the curve (AUC) for RSC3 CD4-binding site mutant compared with RSC3 WT over the course of infection (red line, right-axis) and geometric mean ID_50_ titre on virus indicator panel (black line, left-axis) for SUP033003, SAR032004 and SUT036022, respectively.

## Discussion

Here, we investigated the bnAb responses in 50 ART-naive individuals from the control arm of the SPARTAC trial [31], a primary HIV-1 infection cohort. Similar to other HIV-1 cohort studies [36,40,42,43], plasma neutralization correlated with both duration of HIV-1 infection and viral load, suggesting that both extended levels of antigenic stimulation through chronic infection and high levels of viral antigen are required for the extensive somatic hypermutation that is a characteristic of many HIV-1 bnAbs. Alternatively, these factors increase the probability of activating the germline BCRs that can subsequently develop into bnAbs. When bnAb development was analysed longitudinally (Fig. 3), heterologous neutralization first arose between 50 and 180 weeks postinfection and neutralization breadth (i.e. neutralizing five of six viruses in the indicator panel) arose between 132 and 204 weeks post infection or 24–180 weeks following heterologous neutralization similar to other studies of bnAb development in adults [3,36,39]. These data confirm that early bnAb development is not common (although this has been observed in some infants [44,45]), and is not associated with clinical outcome. In contrast to other studies [3,36,40,41], CD4 levels at both recruitment (<6 months post infection) and the time of bnAb measurement did not negatively correlate with neutralization score. There was, however, a strong negative correlation between viral load and CD4 levels (Fig. 1j), which may suggest that reduced CD4 count is a consequence of high viral replication rather than contributing to bnAb development. Not all individuals with high viral load or long duration of HIV-1 infection developed a bnAb response indicating that other factors are important for bnAb induction.

Due to the relatively small size and the nature of the individuals making up the SPARTAC cohort, it is difficult to tease apart the impact of individual factors, such as HIV-1 subtype, gender, transmission route and location on neutralization score. However, statistically higher neutralization scores were observed in UK donors compared with South African donors. Although not statistically significant, we observed proportionally more male than female participants with neutralization scores above 2.0. In previous analyses of bnAb responses in injecting drug users (IDU) from both the Amsterdam and Swiss cohorts, a significantly lower prevalence of bnAb responses was observed in female compared with male participants [46]. Further, across the whole Swiss cohort, female donors showed slightly lower neutralization breadth than male donors [40]. However, as the route of transmission for male and female participants predominantly differed for members of the SPARTAC cohort, it cannot be concluded that gender alone is responsible for this difference. Black ethnicity has recently been reported to be a strong correlate for bnAb induction in the Swiss cohort [40,42]; however, this information was not available for all SPARTAC participants.

Within the SPARTAC cohort, there is a trend towards higher neutralization scores following a subtype B HIV-1 infection compared with subtype C infection. Conflicting associations between bnAb induction and HIV-1 subtype have been previously reported within other cohorts of HIV-1-infected individuals [36,40]. For example, no difference in bnAb induction was observed between subtype B and nonsubtype B donors in the Swiss cohort [40] yet a strong correlation between HIV-1 subtype C infection and neutralization score was observed in the IAVI Protocol C cohort [36] (although the significance was reduced when considered in a multivariate analysis).

Similar to previous cohort studies, the bnAb activity in SPARTAC donors predominantly mapped to one or two bnAb epitopes. We note that subtle changes in neutralization sensitivity for mutations at several bnAb epitopes were observed in some plasma (Tables S3--S8). The nAbs may also contribute to the overall plasma neutralization breadth and potency. bnAbs targeting glycan-dependent epitopes (V3/N332, N160/V2, and interface epitopes) were particularly prevalent in the SPARTAC cohort [8 of the top 13 donors (62%)]. The number of donors in the SPARTAC cohort that developed bnAbs was too small to find any meaningful differences between HIV-1 subtype and bnAb epitope. When comparing the kinetics of bnAb development with the specificity of the bnAb response, we see that for the donors that target the V3/N332 and N160/V2 epitopes, induction of heterologous neutralization coincides with bnAbs targeting the glycan epitopes. The bnAb responses then broaden incrementally over time as the infection proceeds. This trend in bnAb development was previously seen in donors PC76 from IAVI protocol C [17] and CAP177 and CAP314 from the CAPRISA cohort [47] who developed N332/V3 bnAbs, and donor PC64 from the IAVI protocol C cohort who developed N160/V2 bnAbs [48]. The role that more strain-specific nAbs play in development of bnAb lineages should be investigated in future co-evolution studies.

Although CD4-binding site Abs were common in the SPARTAC cohort (38% of donors showed differential binding to the RSC3 and RSC3 mutant probe to some extent in ELISA), the development of CD4-binding site bnAbs was rare. In the IAVI Protocol C cohort, CD4-binding site Abs took longer to develop than the N332/V3 and N160/V2 bnAbs, presumably because of the very high level of somatic hypermutation required for neutralization breadth [36]. Indeed, Abs with differential binding to the RSC3 protein and CD4-binding site knockout were observed prior to heterologous neutralization suggesting higher mutation levels/antigen stimulation are required for CD4-binding site-targeted nAbs to acquire neutralization breadth (Fig. 4).

In conclusion, we show that in the SPARTAC cohort, development of plasma neutralization breadth and potency correlates with duration of HIV infection and high viral loads. We identify individuals for which more in-depth studies on antibody--viral co-evolution will be conducted to better understand how bnAbs arise during natural infection at the molecular level.

## Acknowledgements

We thank Carl Graham for helpful discussions and critical reading of the manuscript. We thank Dr Christine Mant and Dr John Cason of the King's College London Infectious Diseases Biobank for providing plasma samples. We thank all the participants and staff at all the sites participating in the SPARTAC trial. The SPARTAC Trial Investigators: Trial Steering Committee: Independent Members – A. Breckenridge (Chair), P. Clayden, C. Conlon, F. Conradie, J. Kaldor∗, F. Maggiolo, F. Ssali, Country Principal Investigators- D.A. Cooper, P. Kaleebu, G. Ramjee, M. Schechter, G. Tambussi, J.M. Miro, J. Weber. Trial Physician: S. Fidler. Trial Statistician: A. Babiker. Data and Safety Monitoring Committee (DSMC): T. Peto (Chair), A. McLaren (in memoriam), V. Beral, G. Chene, J. Hakim. Co-ordinating Trial Centre: Medical Research Council Clinical Trials Unit, London (A. Babiker, K. Porter, M. Thomason, F. Ewings, M. Gabriel, D. Johnson, K. Thompson, A. Cursley∗, K. Donegan∗, E. Fossey∗, P. Kelleher∗, K. Lee∗, B. Murphy∗, D. Nock∗). Central Immunology Laboratories and Repositories: The Peter Medawar Building for Pathogen Research, University of Oxford, UK (R. Phillips, J. Frater, L. Ohm Laursen∗, N. Robinson, P. Goulder, H. Brown). Central Virology Laboratories and Repositories: Jefferiss Trust Laboratories, Imperial College, London, UK (M. McClure, D. Bonsall∗, O. Erlwein∗, A. Helander∗, S. Kaye, M. Robinson, L. Cook∗, G. Adcock∗, P. Ahmed∗). Clinical Endpoint Review Committee: N. Paton, S. Fidler. Investigators and Staff at Participating Sites: Australia: St Vincents Hospital, Sydney (A. Kelleher), Northside Clinic, Melbourne (R. Moore), East Sydney Doctors, Sydney (R. McFarlane), Prahran Market Clinic, Melbourne (N. Roth), Taylor Square Private Clinic, Sydney (R. Finlayson), The Centre Clinic, Melbourne (B. Kiem Tee), Sexual Health Centre, Melbourne (T. Read), AIDS Medical Unit, Brisbane (M. Kelly), Burwood Rd Practice, Sydney (N. Doong), Holdsworth House Medical Practice, Sydney (M. Bloch), Aids Research Initiative, Sydney (C. Workman). Coordinating Centre in Australia: Kirby Institute University of New South Wales, Sydney (P. Grey, D.A. Cooper, A. Kelleher, M. Law). Brazil: Projeto Praca Onze, Hospital Escola Sao Francisco de Assis, Universidade federal do Rio de Janeiro, Rio de Janeiro (M. Schechter, P. Gama, M. Mercon∗, M. Barbosa de Souza, C. Beppu Yoshida, J.R. Grangeiro da Silva, A. Sampaio Amaral, D. Fernandes de Aguiar, M. de Fatima Melo, R. Quaresma Garrido). Italy: Ospedale San Raffaele, Milan (G. Tambussi, S. Nozza, M. Pogliaghi, S. Chiappetta, L. Della Torre, E. Gasparotto), Ospedale Lazzaro Spallanzani, Roma (G. DOffizi, C. Vlassi, A. Corpolongo). South Africa: Cape Town: Desmond Tutu HIV-1 Centre, Institute of Infectious Diseases, Cape Town (R. Wood, J. Pitt, C. Orrell, F. Cilliers, R. Croxford, K. Middelkoop, L.G. Bekker, C. Heiberg, J. Aploon, N. Killa, E. Fielder, T. Buhler). Johannesburg: The Wits Reproductive Health and HIV-1 Institute, University of Witswatersrand, Hillbrow Health Precinct, Johannesburg (H. Rees, F. Venter, T. Palanee), Contract Laboratory Services, Johannesburg Hospital, Johannesburg (W. Stevens, C. Ingram, M. Majam, M. Papathanasopoulos). Kwazulu-Natal: HIV-1 Prevention Unit, Medical Research Council, Durban (G. Ramjee, S. Gappoo, J. Moodley, A. Premrajh, L. Zako). Uganda: Medical Research Council/Uganda Virus Research Institute, Entebbe (H. Grosskurth, A. Kamali, P. Kaleebu, U. Bahemuka, J. Mugisha∗, H.F. Njaj∗). Spain: Hospital Clinic-IDIBAPS, University of Barcelona, Barcelona (J.M. Miro, M. Lopez-Dieguez∗, C. Manzardo, J. Ambrosioni, J.A. Arnaiz, T. Pumarola, M. Plana, M. Tuset, M.C. Ligero, M.T. Garcia, T. Gallart, J.M. Gatell). UK and Ireland: Royal Sussex County Hospital, Brighton (M. Fisher, K. Hobbs, N. Perry, D. Pao, D. Maitland, L. Heald), St James's Hospital, Dublin (F. Mulcahy, G. Courtney, S. O’Dea, D. Reidy), Regional Infectious Diseases Unit, Western General Hospital and Genitourinary Dept, Royal Infirmary of Edinburgh, Edinburgh (C. Leen, G. Scott, L. Ellis, S. Morris, P. Simmonds), Chelsea and Westminster Hospital, London (B. Gazzard, D. Hawkins, C. Higgs), Homerton Hospital, London (J. Anderson, S. Mguni), Mortimer Market Centre, London (I. Williams, N. De Esteban, P. Pellegrino, A. Arenas-Pinto, D. Cornforth∗, J. Turner∗), North Middlesex Hospital (J. Ainsworth, A. Waters), Royal Free Hospital, London (M. Johnson, S. Kinloch, A. Carroll, P. Byrne, Z. Cuthbertson), Barts & the London NHS Trust, London (C. Orkin, J. Hand, C. De Souza), St Marys Hospital, London (J. Weber, S. Fidler, E. Hamlyn, E. Thomson∗, J. Fox∗, K. Legg, S. Mullaney∗, A. Winston, S. Wilson, P. Ambrose), Birmingham Heartlands Hospital, Birmingham (S. Taylor, G. Gilleran). Imperial College Trial Secretariat: S. Keeling, A. Becker. Imperial College DSMC Secretariat: C. Boocock.

(Asterisk (∗) indicates that the members left the study team before the trial ended.)

Author contributions: K.J.D., J.F., L.A.G., I.H. designed the study. L.A.G., I.H. and F.D. performed experiments. K.J.D., L.A.G. and I.H. conducted data analysis. P.K., M.S., G.T., J.W., J.M.M., R.P., A.B., D.A.C., M.F., G.R., S.F., J.F., J.F. and SPARTAC Investigators set up the SPARTAC cohort. K.J.D., J.F., I.H. and L.A.G. wrote the manuscript with input from the remaining authors.

Funding: This project has received funding from Wellcome (069598/Z/02/Z), European Union's Horizon 2020 Research and Innovation program under grant agreement no. 681137 (to K.J.D. and I.H.), the Medical Research Council (MRC) (to K.J.D. [MR/K024426/1]), the, Rosetrees Trust (to K.J.D., M686) and Fondation Dormeur, Vaduz (to K.J.D). This research was funded/supported by the National Institute for Health Research (NIHR) Biomedical Research Centre based at Guy's and St Thomas’ NHS Foundation Trust and King's College London and/or the NIHR Clinical Research Facility. The views expressed are those of the author(s) and not necessarily those of the NHS, the NIHR or the Department of Health. L.A.G. were supported by the King's Bioscience Institute and the Guy's and St Thomas’ Charity Prize Ph.D. Programme in Biomedical and Translational Science. J.M.M. received a personal 80 : 20 research grant from Institut d’Investigacions Biomèdiques August Pi i Sunyer (IDIBAPS), Barcelona, Spain, during 2017–2021.

### Conflicts of interest

There are no conflicts of interest.

## Supplementary Material

Supplemental Digital Content
