# Supplementary material for: Broadly neutralizing antibody responses in the longitudinal primary HIV-1 infection Short Pulse Anti-Retroviral Therapy at Seroconversion cohort
Source: AIDS. 2021 Jun 1;35(13):2073–84. doi: 10.1097/QAD.0000000000002988 (PMC8505148; doi:10.1097/QAD.0000000000002988)
Supplement: Supplemental Digital Content [file aids-35-2073-s001.docx]

**Broadly neutralizing antibody responses in the longitudinal primary HIV-1 infection SPARTAC cohort**

Running head: Neutralizing antibodies in SPARTAC cohort

Luke A. Granger,^1,15,16^ Isabella Huettner,^1,16^ Franka Debeljak^1^, Pontiano Kaleebu,^2^ Mauro Schechter,^3^ Giuseppe Tambussi,^4^ Jonathan Weber,^5^ Jose M. Miro,^6^ Rodney Phillips,^7^ Abdel Babiker,^8^ David A. Cooper,^9,17^ Martin Fisher,^10,17^ Gita Ramjee,^11,17^ Sarah Fidler,^12^ John Frater,^13^ Julie Fox,^1,14^ Katie J. Doores^1^* and the SPARTAC Trial Investigators

**Supplemental Tables S1-S6.**

**Table S1: Neutralization of 6-virus panel and clinical data for control arm of SPARTAC cohort.** Neutralization breadth was measured on a cross-clade 6-virus indicator panel and this data was used to calculate a neutralization score (Neut Score, see methods). The ID_50_ values are colour coded based on potency. Data also reported includes weeks post recruitment, geometric mean ID_50_, sex, transmission route (HSW, MSM, MSW), location (UK, Australia (AU), Italy (IT), Brazil (BR), South Africa (SA)), HIV-1 clade, viral load (RNA copies/mL) and CD4 count (per mL) at recruitment, setpoint and the time neutralization was measured.

**Table S2: Multivariate analysis.** A) Correlation of neutralization score versus week post infection (WPI), time in years to initiation of anti-retroviral therapy (ART) and logarithmic viral load at neutralization measurement and at recruitment to trial. Reported are Estimate, Standard (Std.) error, p- and *r^2^-*vales for univariate and multivariate analysis. B) Correlation of neutralization score versus WPI and logarithmic viral load at neutralization measurement. Reported are Estimate, Standard (Std.) error, p- and *r^2^*-vales for univariate and multivariate analysis.

A

B

**Table S3: Epitope mapping for glycan-dependant bnAb epitopes.** Fold changes in ID_50_ for mutant viruses compared to wild-type virus are reported. + indicates neutralization of one virus decreased 3-5 fold, ++ indicates $\geq$2 viurses decreased 3-5-fold, +++ indicates $\geq$5-fold decrease for at least 2 viruses with glycan site deletion.

**Table S4: MPER peptide competition for neutralization.** Fold-decrease in plasma neutralization (ID_50_) when competed with MPER peptide. + indicates a decrease in plasma neutralization potency of $\geq$3-fold when competed with soluble MPER peptide. The viruses used in this assay were selected based upon the neutralization sensitivity in plasma.

**Table S5: RSC3 binding to determine CD4 binding site specificity.** Reported are area under the curve (AUC) of serum binding titres to RSC3, RSC3Δ371I and RSC3Δ371I P363N and the AUC-ratio of RSC3 and RSC3Δ371I P363N. AUCs smaller than the AUC of VRC01 to RSC3Δ371I P363N were set to 50 for simplicity. Binding intensities of sera at 1:50 serum dilution to RSC3 were related to 2G12 binding at 20 µg/mL. RSC3 (VRC01-like): If the ratio between the area under the curve (AUC) for RSC3 /RSC3Δ371I P363N is 2-3 (+/-), 3-8 (+) and >8 (++). RSC3 (no-differential): If the ratio between AUC of RSC3/RSC3Δ371I/P363N) is $\leq$1.8 and the strength is dependent on percentage of 2G12-binding (20 µg/mL) at 1:50 serum-dilution: ++ is $\geq$50%, + is <50%, but $\geq$25%, +/- is <25%.

**Table S6: RSC3 competition for neutralization.** Reported are fold-decreases in ID50 when plasma are competed with RSC3 and RSC3Δ371I/P363N, as well as the ratio between ID50 fold changes for each RSC3 and RSC3Δ371I/P363N. RSC3 competition (VRC01-like): + if the neutralization is decreased by $\geq$3 fold for RSC3 but not RSC3Δ371I/P363N. RSC3 competition (non-differential): + if the neutralization is decreased by $\geq$3 fold for both RCS3 and RSC3Δ371I/P363N.
